# Variation in Label-Rate Insecticide Efficacy and Control Failure Risk Among Brazilian Populations of *Spodoptera frugiperda* (Lepidoptera: Noctuidae)

**DOI:** 10.3390/insects17080825

**Published:** 2026-08-10

**Authors:** Zanandra Z. Tamiosso, Daniela N. Godoy, Ramon B. Palharini, Jéssica L. S. Grzybowski, Marylia P. Cargnin, Venicius E. Pretto, Arthur Dallanora, Josemar Foresti, Dionei S. Muraro, Paulo R. da Silva, Oderlei Bernardi

**Affiliations:** 1 Department of Plant Protection, Federal University of Santa Maria (UFSM), Roraima Avenue 1000, Santa Maria 97105-900, RS, Brazil; zanandrazt@gmail.com (Z.Z.T.); godoyufsm@yahoo.com (D.N.G.); rbpalharini@hotmail.com (R.B.P.); jessica.grzybowski@acad.ufsm.br (J.L.S.G.); maryliapcargnin@gmail.com (M.P.C.); veniciuspretto@outlook.com (V.E.P.); arthurdallanora3@gmail.com (A.D.); 2 Corteva Agriscience, Barueri 06454-080, SP, Brazil; josemar.foresti@corteva.com (J.F.); dionei.schmidtmuraro@corteva.com (D.S.M.); paulor.silva@corteva.com (P.R.d.S.)

**Keywords:** fall armyworm, maize, chemical control, resistance, control efficacy

## Abstract

*Spodoptera frugiperda* (Lepidoptera: Noctuidae) is a key insect pest of maize in Brazil. However, the evolution of insecticide resistance has raised concerns about the risk of control failures under field conditions. This study assessed insecticide efficacy and estimated the risk of control failure in Brazilian populations of *S. frugiperda* exposed to selected insecticides. Nine field populations were collected from representative maize-producing regions of Brazil during the 2024/2025 and 2025/2026 seasons. Early third-instar (L3) larvae from these populations were exposed to the highest label rate of ten insecticides representing different modes of action in maize leaf bioassays. Mortality was assessed 96 h after exposure, and the risk of control failure was based on the efficacy threshold of 80% mortality. Significant effects of population, insecticide, and the population × insecticide interaction were detected, indicating variation in susceptibility among the sampled populations. Methoxyfenozide + spinetoram, spinosad, chlorfenapyr, and spinetoram consistently caused high mortality (>81%) and were associated with low risk of control failure across all populations. In contrast, chlorantraniliprole exhibited reduced efficacy with mortality below the expected efficacy threshold in most populations and a high risk of control failure. Population-dependent variation in insecticide efficacy was also evident for metaflumizone, emamectin benzoate, thiodicarb, indoxacarb, and methoxyfenozide, with corresponding variation in the risk of control failure. These findings show that insecticide efficacy and the risk of control failure depend on the specific insecticide–population combination and provide valuable information to support decision-making regarding insecticide selection and the rotation of modes of action within insect resistance management (IRM) and integrated pest management (IPM) programs.

## Simple Summary

The fall armyworm (*Spodoptera frugiperda*) is a key pest of maize in Brazil. The use of insecticides is essential for controlling this pest in maize, but differences in susceptibility among populations can reduce insecticide effectiveness. In this study, we evaluated insecticides against *S. frugiperda* populations collected from maize-producing regions of Brazil. Spinosad, spinetoram, chlorfenapyr, and the mixture of methoxyfenozide + spinetoram consistently provided high levels of control across all populations. In contrast, chlorantraniliprole showed low effectiveness in most populations, evidencing a high risk of control failure. Other insecticides, including metaflumizone, emamectin benzoate, thiodicarb, indoxacarb, and methoxyfenozide, showed variable performance among the populations evaluated. These findings identify insecticide–population combinations associated with an increased risk of control failure and highlight the importance of considering population-dependent variation in susceptibility when selecting insecticides for *S. frugiperda* control in maize.

## 1. Introduction

The fall armyworm, *Spodoptera frugiperda* (J. E. Smith, 1797) (Lepidoptera: Noctuidae), is one of the most important insect pests of maize worldwide [1]. In addition to maize, this species exploits a wide range of other cultivated plants and has also been reported causing economic losses in cotton (*Gossypium hirsutum* L.), soybean (*Glycine max* (L.) Merrill), rice (*Oryza sativa* L.), sorghum (*Sorghum bicolor* (L.) Moench), wheat (*Triticum aestivum* L.), and oat (*Avena sativa* L.) [2]. In Brazil, the overlapping cultivation of host crops and the occurrence of multiple maize-growing seasons provide favorable conditions for the occurrence of successive generations throughout the year, contributing to the persistence of large *S. frugiperda* populations across Brazilian agricultural landscapes [3,4].

The management of *S. frugiperda* relies primarily on Bt crops and chemical insecticides [5,6]. However, the continuous cultivation of Bt maize, combined with poor compliance with insect resistance management (IRM) practices, particularly the use of refuge areas, has led to the evolution of resistance to several Bt proteins [7,8,9,10]. As the efficacy of Bt technologies declines, insecticide applications become increasingly important for maintaining effective pest control in maize production systems [11,12].

Despite the importance of chemical control, achieving effective insecticide control of *S. frugiperda* in maize remains challenging. Larvae are often protected within the maize whorl, limiting insecticide exposure, while overlapping generations and continuous infestations frequently require multiple applications during the growing season [13,14]. These factors increase the intensity of insecticide use and have contributed to the evolution of resistance to several insecticide groups commonly used against *S. frugiperda*, including pyrethroids, organophosphates, benzoylureas, spinosyns, diamides, and emamectin benzoate [15,16,17,18,19,20].

The reduction in susceptibility of *S. frugiperda* to insecticides has important agronomic and economic consequences. As field efficacy declines, growers often increase the frequency of insecticide applications or replace selective insecticides with broader-spectrum, more toxic alternatives to maintain satisfactory pest control. These practices increase production costs, intensify selection pressure for resistance, and may adversely affect beneficial arthropods and other non-target organisms [5,12]. Therefore, preserving the efficacy of insecticides with different modes of action, particularly those that are more selective to natural enemies and have lower environmental impact, is essential for the sustainable management of *S. frugiperda*. Accordingly, regular monitoring of insecticide efficacy and the estimated risk of control failure across maize-producing regions is essential to support IRM and integrated pest management (IPM) programs.

To address this issue, this study assessed insecticide efficacy and the estimated risk of control failure among Brazilian populations of *S. frugiperda* collected from representative maize-producing regions. The findings provide practical information to support insecticide selection and the implementation of IRM and IPM strategies for this pest.

## 2. Materials and Methods

### 2.1. Insect Collection and Rearing

**Figure 1:**
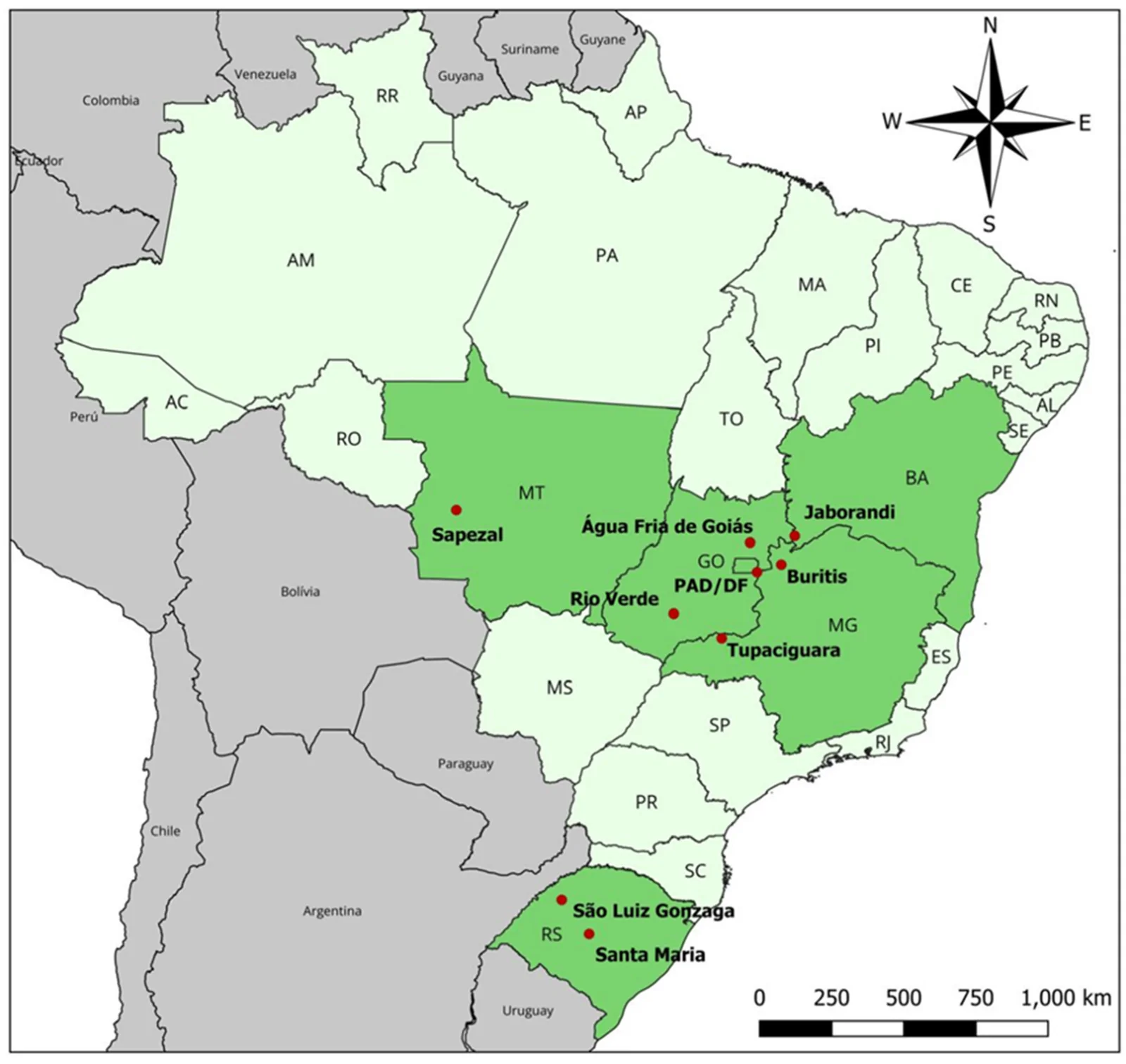
Geographic origin of *S. frugiperda* populations collected from maize fields in Brazil during the 2024/2025 and 2025/2026 growing seasons.

Nine populations of *S. frugiperda* were collected from maize fields across five Brazilian states during the 2024/2025 and 2025/2026 growing seasons (300–1200 larvae per collection site) (Table 1; Figure 1). The collection sites were in major maize grain- and seed-producing regions of Brazil, representing the country’s principal maize production systems, where maize cultivation and insecticide use are intensive. Collections were conducted under permits issued by the Brazilian Ministry of the Environment through the System of Authorization and Information on Biodiversity (SISBIO) (scientific collecting permits No. 65052-18 and 65052-23). After collection, larvae were transported to the laboratory and individually reared in 50 mL plastic cups containing the artificial diet described by Greene et al. [21] until pupation. Approximately 200–1000 field-collected larvae from each population developed into adults. Adults from each population were then transferred to polyvinyl chloride (PVC) cages (20 cm height × 20 cm diameter) lined with white sulfite paper and covered with voile fabric for mating and oviposition (50–80 couples per cage). For each field population, adults were maintained in multiple mating cages, and the offspring from all cages were pooled to establish the F_1_-generation larvae used in the bioassays. In addition to the field populations, a susceptible laboratory population, maintained since 2012 without exposure to insecticides or Bt proteins, was used as the source of susceptible insects.

### 2.2. Insecticides

Ten insecticides representing different modes of action and widely used for the control of *S. frugiperda* in Brazil were selected for this study. Each commercial product was tested at the highest label rate registered for its use in maize (Table 2).

### 2.3. Bioassays

Bioassays were conducted using maize leaves. A non-Bt maize hybrid (Supremo SX7331; Syngenta Seeds, São Paulo, SP, Brazil) was grown under field conditions in Santa Maria, RS, Brazil (29°43′1.25″ S, 53°44′2.48″ W), at a density of four plants/meter. Each treatment plot consisted of five 5 m maize rows spaced 0.45 m apart, resulting in a total plot area of 11.25 m^2^ and containing ~100 plants. Adjacent plots were separated by 10 m buffer zones to minimize spray drift between treatments. Because the field populations were collected at different times throughout the growing season, each population was evaluated independently using the same standardized experimental protocol.

At the V_6_ growth stage, maize plants were sprayed with each insecticide at the highest label rate registered for *S. frugiperda* control in maize (Table 2). Applications were performed using a CO_2_-pressurized backpack sprayer equipped with a 2 m bar, 0.5 m nozzle spacing, and XR110.02 flat-fan nozzles (TeeJet Technologies Co., Glendale Heights, IL, USA), calibrated to deliver 150 L/ha. To prevent cross-contamination among treatments, the spray equipment was thoroughly cleaned between consecutive insecticide applications. Thirty minutes after application, whorl leaves were collected from at least 50 randomly selected plants within each plot, transported to the laboratory, and cut into 12-cm^2^ pieces. This interval allowed the spray deposits to dry while minimizing environmental degradation of insecticide residues before the laboratory bioassays. The leaf pieces were then placed on filter paper over a 2.5% agar–water layer (Dinâmica Química, Indaiatuba, SP, Brazil) in 42-well bioassay plates (BioSupply, São Paulo, SP, Brazil), with two pieces/well. Leaves from untreated plants were used as the control treatment. A single early third-instar (L3) larva (~1 cm in length; head capsule width: ~0.75 mm) was placed in each well. Plates were sealed with their respective plastic lids and maintained in a room at 25 ± 2 °C, 65 ± 10% relative humidity, and a 14:10 h photoperiod.

The laboratory bioassays followed a completely randomized design with six biological replicates per insecticide–population combination. Each biological replicate consisted of 21 third-instar larvae, resulting in a total of 126 larvae evaluated for each insecticide–population combination. Larval mortality was assessed 96 h after exposure, during which the treated leaf material was not replaced. The 96 h assessment period was selected to allow all evaluated insecticides, including the slower-acting methoxyfenozide, to express their full insecticidal activity and ensure consistent comparisons among compounds with different modes of action. Larvae that failed to respond to gentle stimulation with a fine paintbrush were considered dead.

### 2.4. Statistical Analysis

Data were analyzed using generalized linear models (GLMs) with a quasi-binomial error distribution and logit link function. The response variable was specified as the numbers of dead and surviving larvae at the replicate level and was implemented as cbind (dead, alive). Population, insecticide, and their interaction were included as fixed effects. An untreated control was used exclusively for mortality correction and was not included as a treatment in the GLMs. The significance of population, insecticide, and their interaction was assessed using Type II Analysis of Deviance with *F*-tests. When the population × insecticide interaction was significant, only simple effects were interpreted (i.e., comparisons among populations within each insecticide and among insecticides within each population).

The risk of control failure (RCF) was estimated using Equation (1), as proposed by Guedes [22]:

RCF = 100 − [(Observed mortality × 100)/Expected mortality],(1)

where observed mortality corresponds to the Abbott-corrected mortality [23], expressed as a percentage. The expected mortality was set at 80%, corresponding to the minimum efficacy threshold commonly required for insecticide registration in Brazil and widely used to assess insecticide performance [22]. According to Equation (1), RCF was negative whenever observed mortality exceeded the expected mortality threshold (80%). Negative RCF values were set to zero because observed mortality exceeded the expected efficacy threshold. Mortality values below the 80% threshold generated positive RCF values, which quantify the magnitude of the potential control failure relative to the expected efficacy threshold. Estimated marginal means and their associated one-sided contrasts were obtained from the fitted quasi-binomial GLM to test whether Abbott-corrected mortality was lower than the expected efficacy threshold of 80%. The null and alternative hypotheses were H_0_: *p* = 0.80 and H_a_: *p* < 0.80, respectively. To control the family-wise error rate (FWER) across all comparisons, *p*-values were adjusted using the Holm procedure. Differences were considered statistically significant when the Holm-adjusted *p*-value was <0.05. Therefore, evidence of control failure risk was considered present only when Abbott-corrected mortality was significantly lower than the expected efficacy threshold of 80%.

All statistical analyses were performed using R version 4.5.1 [24].

## 3. Results

There were significant effects of population (*F* = 94.41, *df* = 9, *p* < 0.0001), insecticide (*F* = 169.78, *df* = 9, *p* < 0.0001), and the population × insecticide interaction (*F* = 9.45, *df* = 81, *p* < 0.0001). Because the population × insecticide interaction was significant, subsequent interpretation was based on simple-effect comparisons among insecticides within each population and among populations within each insecticide. These analyses showed that insecticide efficacy, and consequently the risk of control failure, depended on the specific population–insecticide combination.

### 3.1. Population-Specific Effects

**Figure 2:**
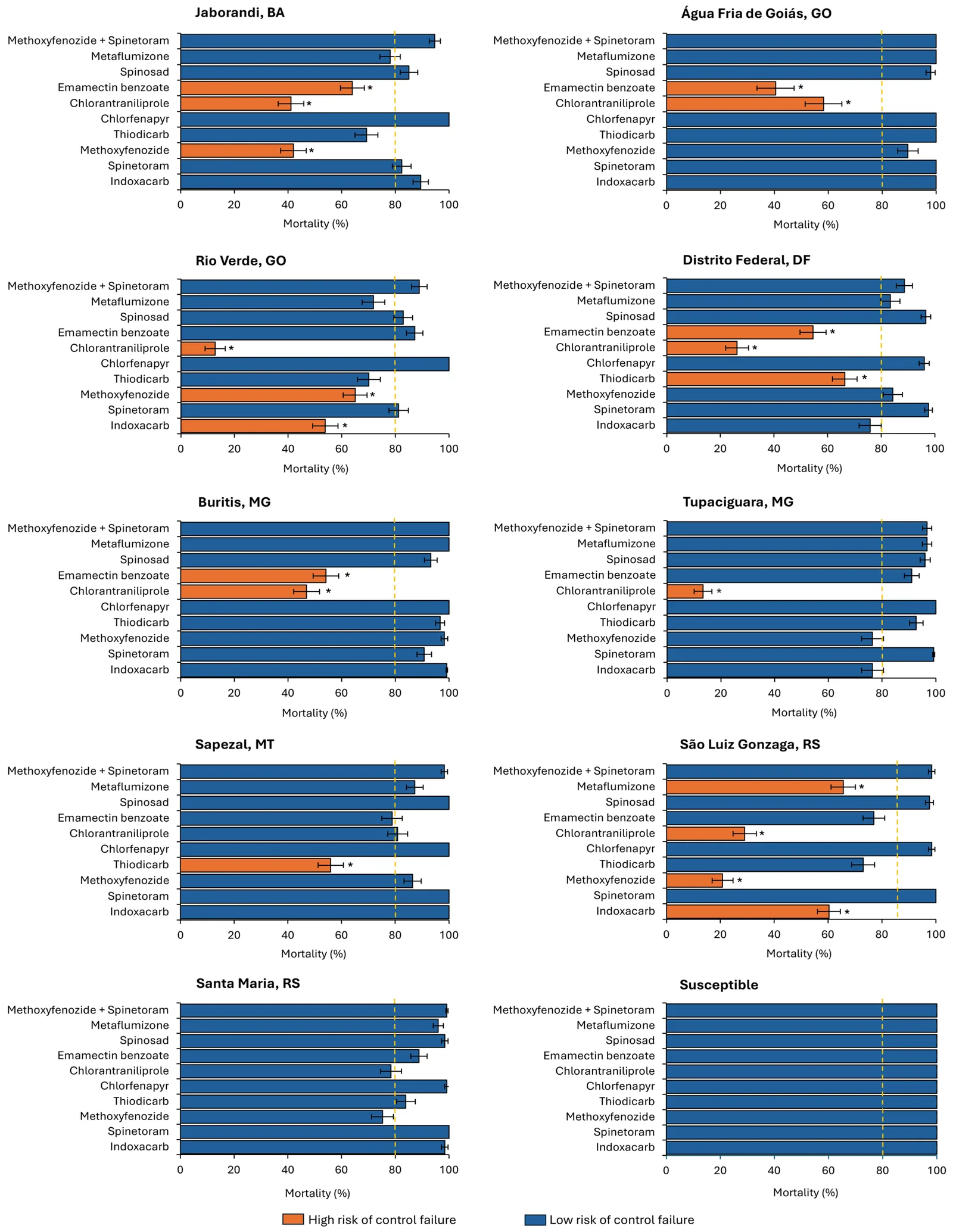
Population-specific mortality (mean ± SE) of Brazilian populations of *S. frugiperda* following exposure to the highest label rates of selected insecticides. Asterisks (*) indicate Abbott-corrected mortality significantly lower than the expected efficacy threshold of 80%, based on one-sided quasi-binomial GLMs with Holm-adjusted *p*-values (*p* < 0.05).

**Figure 3:**
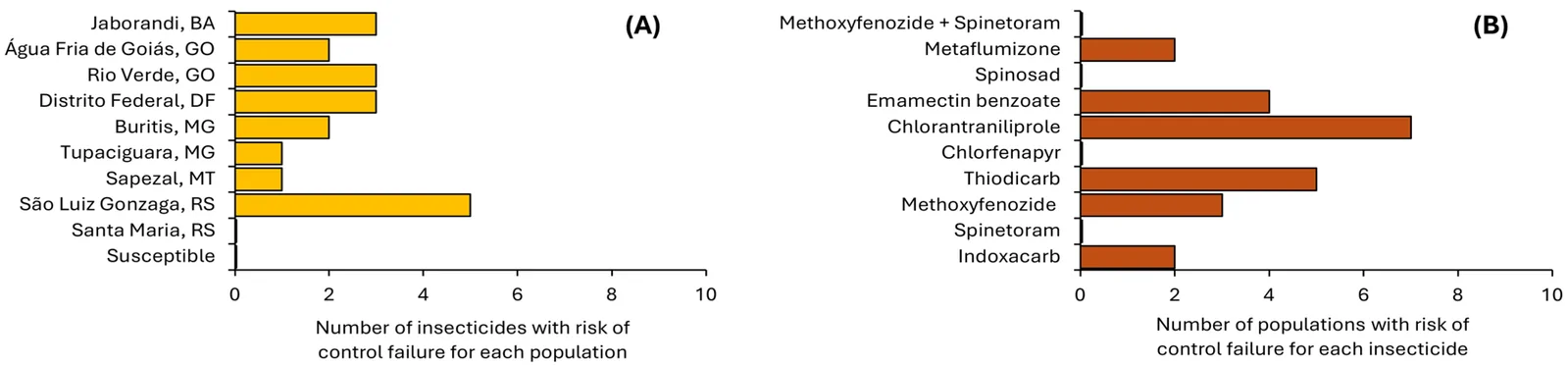
Number of insecticides with risk of control failure for each *S. frugiperda* population (**A**) and number of populations with risk of control failure for each insecticide (**B**). Only populations with mortality significantly lower than the 80% efficacy threshold, based on one-sided quasi-binomial GLMs with Holm-adjusted *p*-values (*p* < 0.05), were considered to be at risk of control failure for a given insecticide.

The *S. frugiperda* populations exhibited substantial variation in their response to the insecticides evaluated (Figure 2). Populations from Água Fria de Goiás–GO, Buritis–MG, Tupaciguara–MG, Sapezal–MT, Distrito Federal–DF, and Santa Maria–RS exhibited the highest mortality (75.8–100%) when exposed to methoxyfenozide + spinetoram, metaflumizone, spinosad, spinetoram, chlorfenapyr, and indoxacarb (Figure 2; Table S1). Accordingly, mortality was not significantly lower than the expected 80% threshold, and the risk of control failure remained low (≤5.2%) for these insecticides (Figure 3A; Table S1). In contrast, chlorantraniliprole provided poor control in most populations. Except for Sapezal–MT (80.9%) and Santa Maria–RS (78.4%), mortality remained below 59%, indicating a high risk of control failure (>27%). In addition, populations from Distrito Federal–DF and Sapezal–MT showed reduced control efficacy with thiodicarb, with mortality ≤66.3% and a corresponding risk of control failure exceeding 17%.

Populations from Jaborandi–BA, Rio Verde–GO, and São Luiz Gonzaga–RS exhibited reduced responses to some insecticides (Figure 2; Table S1). All three populations showed low mortality following exposure to chlorantraniliprole (12.8–41.1%). Reduced mortality following exposure to emamectin benzoate (<64%) was also observed in populations from Jaborandi–BA, Buritis–MG, Distrito Federal–DF, and Água Fria de Goiás–GO, with corresponding high risk of control failure (Table S1). Furthermore, Rio Verde–GO and São Luiz Gonzaga–RS consistently exhibited reduced responses to methoxyfenozide and indoxacarb, with mortality below 65% and a high risk of control failure. Overall, the populations from Jaborandi–BA, Rio Verde–GO, Distrito Federal–DF, and São Luiz Gonzaga–RS were associated with the highest number of insecticides presenting a risk of control failure (Figure 3A; Table S1).

The susceptible reference population showed 100% mortality when exposed to the highest field rate of all insecticides (Figure 2), whereas several field populations exhibited reduced mortality, potentially indicating reduced susceptibility.

### 3.2. Insecticide-Specific Effects

**Figure 4:**
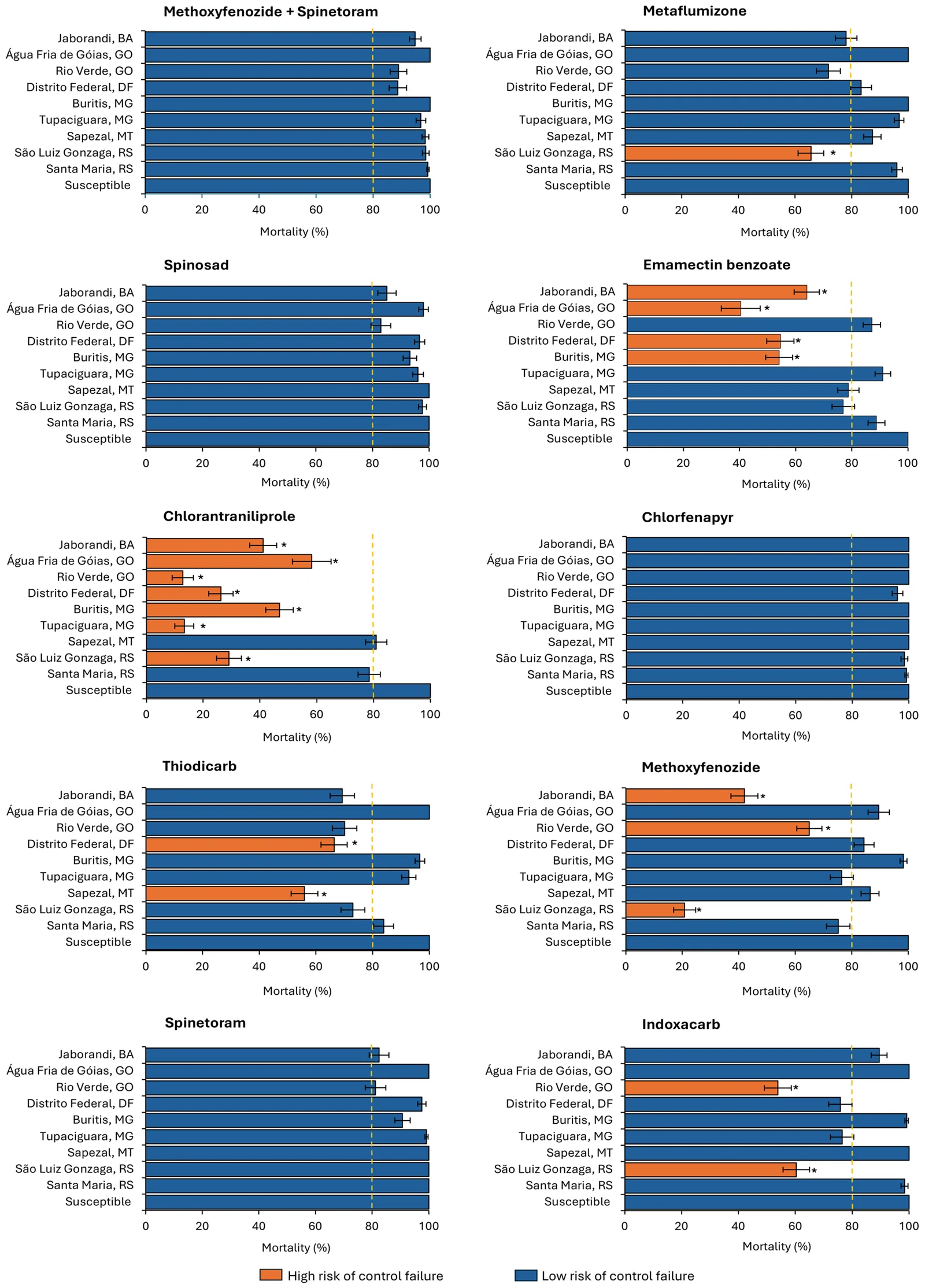
Insecticide-specific mortality (mean ± SE) caused by the highest label rates of selected insecticides in Brazilian populations of *S. frugiperda*. Asterisks (*) indicate Abbott-corrected mortality significantly lower than the expected efficacy threshold of 80%, based on one-sided quasi-binomial GLMs with Holm-adjusted *p*-values (*p* < 0.05).

Methoxyfenozide + spinetoram, spinosad, chlorfenapyr, and spinetoram consistently caused high mortality (>81%) in all *S. frugiperda* populations evaluated (Figure 4; Table S1). Accordingly, these insecticides were associated with a low risk of control failure, as mortality remained above the expected efficacy threshold of 80% (Figure 3B). Metaflumizone also provided high efficacy in most populations (78–100%). However, mortality in the São Luiz Gonzaga–RS population was below 66% and significantly lower than the expected efficacy threshold, indicating a high risk of control failure (Table S1). In contrast, chlorantraniliprole exhibited reduced efficacy in most populations, with mortality below 60% in seven of the nine field populations evaluated. Consequently, this insecticide was associated with the highest risk of control failure, frequently exceeding 40% (Figure 3B and Figure 4; Table S1).

Emamectin benzoate, thiodicarb, indoxacarb, and methoxyfenozide exhibited some variation in efficacy among *S. frugiperda* populations (Figure 4; Table S1). Except for indoxacarb, these insecticides caused relatively low mortality (<70%) in the population from Jaborandi–BA. Reduced mortality was also observed for emamectin benzoate in populations from Água Fria de Goiás–GO, Distrito Federal–DF, and Buritis–MG (<55%); for thiodicarb in populations from Sapezal–MT and Distrito Federal–DF (<67%); and for indoxacarb in populations from Rio Verde–GO and São Luiz Gonzaga–RS (<61%). These reductions in efficacy were associated with high risks of control failure for the corresponding insecticide–population combinations (Figure 4; Table S1).

Overall, the insecticides differed markedly in their efficacy against Brazilian populations of *S. frugiperda*, resulting in distinct population-specific patterns of control failure risk.

## 4. Discussion

The management of *S. frugiperda* in Brazil relies heavily on insecticide applications, making the preservation of insecticide efficacy essential for sustainable pest control. In this context, monitoring insecticide efficacy and identifying insecticide–population combinations associated with an increased risk of control failure are key components of IRM and IPM programs. The present study showed that insecticide efficacy differed among the sampled Brazilian populations of *S. frugiperda*, highlighting the need for continuous monitoring to support region-specific insecticide selection and the implementation of effective IRM practices.

The widespread reduction in chlorantraniliprole efficacy is particularly concerning given the importance of diamides for *S. frugiperda* management in Brazil. Since their introduction, chlorantraniliprole and other diamides have been valued for their high efficacy, selectivity, and favorable environmental profile, resulting in their widespread adoption by growers [25,26]. In Brazil, reduced susceptibility to chlorantraniliprole and flubendiamide, together with increased frequencies of chlorantraniliprole resistance alleles, has been reported in field populations of *S. frugiperda* from major maize-producing regions, particularly in Bahia, during the 2011–2014 and 2016 maize seasons [19,27]. Subsequent studies characterized a chlorantraniliprole-resistant *S. frugiperda* strain exhibiting high levels of resistance and cross-resistance to other diamides, including flubendiamide and cyantraniliprole [19]. Collectively, these studies support the hypothesis that intensive and repeated use of diamides, particularly chlorantraniliprole, has contributed to the selection for resistant individuals in Brazilian populations of *S. frugiperda*. In this species, resistance to diamides appears to be primarily associated with mutations in the ryanodine receptor (RyR), the molecular target of these insecticides [28,29]. Although the present study did not investigate the underlying resistance mechanism, the reduced efficacy of chlorantraniliprole observed here is consistent with previous reports of field-evolved resistance. These findings reinforce the need for proactive IRM strategies to preserve the efficacy of this important insecticide group.

Unlike chlorantraniliprole, methoxyfenozide + spinetoram, spinetoram, spinosad, and chlorfenapyr maintained consistently high efficacy across all *S. frugiperda* populations evaluated, indicating that these insecticides remain effective options for *S. frugiperda* control in Brazil. High susceptibility to chlorfenapyr and low frequencies of resistance alleles were previously reported in Brazilian *S. frugiperda* populations collected from major maize-producing regions between 2016 and 2018 [30]. Although resistance to spinosad and spinetoram has been documented in *S. frugiperda* in Brazil [18,31], the consistently high efficacy observed in the present study suggests that the evaluated populations remain susceptible to these insecticides. Resistance to spinosyns has been associated with enhanced metabolic detoxification mediated by cytochrome P450 monooxygenases and, in some cases, with mutations in the nicotinic acetylcholine receptor α6 (nAChR α6) subunit [32,33]. These results highlight the importance of maintaining regular susceptibility monitoring to preserve the long-term efficacy of spinosyns.

Emamectin benzoate, metaflumizone, thiodicarb, methoxyfenozide, and indoxacarb showed population-dependent responses, indicating differences in susceptibility and estimated risk of control failure among the sampled populations. For emamectin benzoate, field-evolved resistance has been documented in Brazilian populations of *S. frugiperda* since 2019, with the greatest reductions in susceptibility reported in populations from the Central-West and Northeast maize-producing regions [20]. This is consistent with our results, in which the populations sampled from these regions exhibited the highest risk of control failure. In contrast, a large-scale study conducted between 2017 and 2020 reported low variation in the susceptibility of Brazilian *S. frugiperda* populations to the sodium channel blockers indoxacarb (4.6-fold) and metaflumizone (2.6-fold), together with low frequencies of resistance alleles, indicating that susceptibility to both insecticides remained largely preserved during this period [6]. Similarly, Amado [34] reported low variation in susceptibility to methoxyfenozide among Brazilian populations of *S. frugiperda* collected during the second maize season of 2016 and the first maize season of 2017. However, several field populations exhibited increased survival at the diagnostic concentration during the 2017 first maize season. Where investigated, resistance to emamectin benzoate, indoxacarb, and methoxyfenozide in *Spodoptera* species has been associated with enhanced metabolic detoxification [35,36,37], whereas the mechanisms underlying resistance to metaflumizone and thiodicarb remain poorly understood. In agreement with these previous studies, risks of control failure for indoxacarb, metaflumizone, methoxyfenozide, and thiodicarb were detected in only a limited number of populations, suggesting that reduced susceptibility is currently restricted to a limited number of populations and may reflect differences in local selection pressure. Future studies integrating field-level insecticide use history with resistance monitoring will help clarify the extent to which local selection pressure contributes to the observed variation in susceptibility.

Overall, the variation in insecticide efficacy observed among Brazilian populations of *S. frugiperda* demonstrates that insecticide performance depends on the specific insecticide–population combination. These findings highlight the importance of incorporating regional susceptibility monitoring into IRM programs to guide region-specific insecticide selection and preserve the long-term efficacy of available insecticides.

## 5. Conclusions

The efficacy of insecticides evaluated against the Brazilian field populations of *S. frugiperda* evaluated varied among populations and insecticides. Under the current susceptibility scenario, chlorfenapyr, spinetoram, spinosad, and methoxyfenozide + spinetoram consistently provided effective control and showed a low risk of control failure. In contrast, chlorantraniliprole exhibited reduced efficacy in most populations and was associated with a high risk of control failure. Variation in insecticide efficacy among the sampled populations was observed for metaflumizone, emamectin benzoate, thiodicarb, indoxacarb, and methoxyfenozide, indicating population-dependent responses. These findings provide an updated assessment of insecticide efficacy among Brazilian field populations of *S. frugiperda* and support evidence-based insecticide selection and IRM strategies aimed at preserving insecticide efficacy and reducing the risk of control failure.

## Figures and Tables

**Table 1:** Collection sites of Brazilian populations of *S. frugiperda* used to assess the risk of insecticide control failure.

Population	Latitude	Longitude	Date of Collection
Rio Verde, GO	17°35′45″ S	50°31′30″ W	February 2025
Tupaciguara, MG	18°31′46″ S	48°42′14″ W	April 2025
Santa Maria, RS	29°43′01″ S	53°44′02″ W	April 2025
PAD/DF, DF	16°01′28″ S	47°21′56″ W	April 2025
Jaborandi, BA	14°39′25″ S	45°56′08″ W	May 2025
Água Fria de Goiás, GO	14°54′18″ S	47°37′40″ W	June 2025
Buritis, MG	15°44′18″ S	46°26′45″ W	June 2025
São Luiz Gonzaga, RS	28°24′53″ S	54°46′22″ W	October 2025
Sapezal, MT	13°40′16″ S	58°46′19″ W	January 2026

**Table 2:** Insecticides and corresponding label rates used to assess insecticide efficacy and the estimated risk of control failure in Brazilian populations of *S. frugiperda*.

Active Ingredient (IRAC MoA) ^1^	Trade Name	A.I. (%)	Company/Manufacturer	mL or g/ha ^2^	g a.i./ha ^2^
Methoxyfenozide (18)	Intrepid^®^ 240 SC	24	CTVA Proteção de Cultivos Ltda., Barueri, SP, Brazil	180	43.2
Chlorantraniliprole (28)	Premio^®^	20	FMC Química do Brasil Ltda, Campinas, SP, Brazil	125	25
Thiodicarb (1A)	Larvin 800 WG	80	Bayer S.A., São Paulo, SP, Brazil	150	120
Spinetoram (5)	Exalt^®^	12	CTVA Proteção de Cultivos Ltda., Barueri, SP, Brazil	100	12
Spinosad (5)	Tracer^®^	48	CTVA Proteção de Cultivos Ltda., Barueri, SP, Brazil	100	48
Emamectin benzoate (6)	Proclaim^®^ 50	5	Syngenta Proteção de Cultivos Ltda., São Paulo, SP, Brazil	300	15
Indoxacarb (22A)	Avatar^®^	15	FMC Química do Brasil Ltda, Campinas, SP, Brazil	400	60
Metaflumizone (22B)	Verismo^®^	24	BASF S.A., São Paulo, SP, Brazil	1000	240
Chlorfenapyr (13)	Pirate^®^	24	BASF S.A., São Paulo, SP, Brazil	1000	240
Methoxyfenozide (18) + Spinetoram (5)	Intrepid^®^ 240 SC + Exalt^®^	24 + 12	CTVA Proteção de Cultivos Ltda., Barueri, SP, Brazil	180 + 100	43.2 + 12

^1^ IRAC, Insecticide Resistance Action Committee; MoA, mode of action. ^2^ Label rate of the commercial product (mL or g/ha) tested in maize leaf bioassays and the corresponding rate expressed as grams of active ingredient (g a.i./ha).

## Data Availability

The original contributions presented in this study are included in the article/Supplementary Materials. Further inquiries can be directed to the corresponding author.

## Supplementary Materials

The following supporting information can be downloaded at: https://www.mdpi.com/article/10.3390/insects17080825/s1, Table S1: Mortality (% ± SE) and risk of control failure (%) of *S. frugiperda* populations exposed to the highest recommended label rates of the evaluated insecticides.
