# Supplementary material for: Variation in Label-Rate Insecticide Efficacy and Control Failure Risk Among Brazilian Populations of Spodoptera frugiperda (Lepidoptera: Noctuidae)
Source: Insects. 2026 Aug 10;17(8):825. doi: 10.3390/insects17080825 (PMC13513220; doi:10.3390/insects17080825)
Supplement: Supplementary file 1 [file insects-17-00825-s001.zip › insects-4470999-supplementary.pdf]

Table S1. Mortality (% ± SE) and risk of control failure (%) of *S. frugiperda* populations exposed to the highest recommended label rates of the evaluated insecticides.

Population	Mortality (%) [Risk of Control Failure (%)][1]				
	Methoxyfenozide + Spinetoram	Metaflumizone	Spinosad	Emamectin benzoate	Chlorantraniliprole
Jaborandi, BA	94.7 ± 2.2 [0.0]	78.0 ± 3.7 [2.5]	85.1 ± 3.2 [0.0]	63.9 ± 4.4 [20.0]*	41.1 ± 4.7 [48.6]*
Água Fria de Goiás, GO	100.0 ± 0.0 [0.0]	100.0 ± 0.0 [0.0]	97.9 ± 1.6 [0.0]	40.4 ± 6.8 [49.4]*	58.2 ± 6.7 [27.2]*
Rio Verde, GO	88.8 ± 2.8 [0.0]	71.7 ± 4.1 [10.2]	82.9 ± 3.4 [0.0]	87.1 ± 3.0 [0.0]	12.8 ± 3.7 [83.9]*
Distrito Federal, DF	88.6 ± 3.0 [0.0]	83.3 ± 3.6 [0.0]	96.6 ± 1.7 [0.0]	54.5 ± 4.8 [31.8]*	26.3 ± 4.2 [67.2]*
Buritis, MG	100.0 ± 0.0 [0.0]	100.0 ± 0.0 [0.0]	93.2 ± 2.3 [0.0]	54.1 ± 4.7 [32.3]*	46.8 ± 4.8 [41.3]*
Tupaciguara, MG	96.7 ± 1.6 [0.0]	96.7 ± 1.7 [0.0]	96.0 ± 1.8 [0.0]	91.0 ± 2.7 [0.0]	13.3 ± 3.3 [83.3]*
Sapezal, MT	98.3 ± 1.1 [0.0]	87.2 ± 3.0 [0.0]	100.0 ± 0.0 [0.0]	78.8 ± 3.7 [1.5]	80.9 ± 3.6 [0.0]
São Luiz Gonzaga, RS	98.4 ± 1.1 [0.0]	65.6 ± 4.5 [18.0]*	97.6 ± 1.4 [0.0]	76.9 ± 4.0 [3.8]	29.0 ± 4.3 [63.7]*
Santa Maria, RS	99.2 ± 0.8 [0.0]	95.9 ± 1.8 [0.0]	98.4 ± 1.1 [0.0]	88.8 ± 2.9 [0.0]	78.4 ± 3.9 [2.0]
Susceptible	100.0 ± 0.0 [0.0]	100.0 ± 0.0 [0.0]	100.0 ± 0.0 [0.0]	100.0 ± 0.0 [0.0]	100.0 ± 0.0 [0.0]
	Chlorfenapyr	Thiodicarb	Methoxyfenozide	Spinetoram	Indoxacarb
Jaborandi, BA	100.0 ± 0.0 [0.0]	69.2 ± 4.2 [13.4]	42.0 ± 4.7 [47.5]*	82.4 ± 3.4 [0.0]	89.5 ± 2.7 [0.0]
Água Fria de Goiás, GO	100.0 ± 0.0 [0.0]	100.0 ± 0.0 [0.0]	89.5 ± 3.7 [0.0]	100.0 ± 0.0 [0.0]	100.0 ± 0.0 [0.0]
Rio Verde, GO	100.0 ± 0.0 [0.0]	70.0 ± 4.2 [12.3]	64.9 ± 4.4 [18.8]*	81.2 ± 3.6 [0.0]	53.8 ± 4.7 [32.6]*
Distrito Federal, DF	96.0 ± 1.8 [0.0]	66.3 ± 4.5 [17.0]*	84.3 ± 3.5 [0.0]	97.5 ± 1.4 [0.0]	75.8 ± 4.1 [5.2]
Buritis, MG	100.0 ± 0.0 [0.0]	96.6 ± 1.6 [0.0]	98.2 ± 1.2 [0.0]	93.2 ± 2.3 [0.0]	99.1 ± 0.8 [0.0]
Tupaciguara, MG	100.0 ± 0.0 [0.0]	92.7 ± 2.4 [0.0]	76.4 ± 4.0 [4.5]	99.1 ± 0.8 [0.0]	76.4 ± 4.0 [4.6]
Sapezal, MT	100.0 ± 0.0 [0.0]	55.9 ± 4.6 [30.0]*	86.4 ± 3.1 [0.0]	100.0 ± 0.0 [0.0]	100.0 ± 0.0 [0.0]
São Luiz Gonzaga, RS	98.4 ± 1.1 [0.0]	73.0 ± 4.2 [8.7]	20.8 ± 3.8 [74.0]*	100.0 ± 0.0 [0.0]	60.3 ± 4.6 [24.6]*
Santa Maria, RS	99.2 ± 0.8 [0.0]	83.8 ± 3.5 [0.0]	75.2 ± 4.0 [6.0]	100.0 ± 0.0 [0.0]	98.4 ± 1.1 [0.0]
Susceptible	100.0 ± 0.0 [0.0]	100.0 ± 0.0 [0.0]	100.0 ± 0.0 [0.0]	100.0 ± 0.0 [0.0]	100.0 ± 0.0 [0.0]

[1] Asterisks (*) indicate Abbott-corrected mortality (mean ± SE) significantly lower than the expected efficacy threshold of 80%, based on one-sided quasi-binomial GLMs with Holm-adjusted p-values ($p < 0.05$).
